# Modelling elephant corridors over two decades reveals opportunities for conserving connectivity across a large protected area network

**DOI:** 10.1371/journal.pone.0292918

**Published:** 2023-10-13

**Authors:** Richard A. Giliba, Christian Kiffner, Pascal Fust, Jacqueline Loos

**Affiliations:** 1 The Nelson Mandela African Institution of Science and Technology, School of Life Sciences and Bio-Engineering, Arusha, Tanzania; 2 Institute of Ecology, Leuphana University Lüneburg, Lüneburg, Germany; 3 Department of Human Behavior, Ecology and Culture, Max Planck Institute for Evolutionary Anthropology, Leipzig, Germany; 4 Junior Research Group Human-Wildlife Conflict & Coexistence, Leibniz Centre for Agricultural Landscape Research (ZALF), Research Area Land-use and Governance, Müncheberg, Germany; 5 Social-Ecological Systems Institute, Leuphana University Lüneburg, Lüneburg, Germany; Instituto Federal de Educacao Ciencia e Tecnologia Goiano - Campus Urutai, BRAZIL

## Abstract

Protected area (PA) connectivity is pivotal for the persistence of wide-ranging wildlife species, but is challenged by habitat loss and fragmentation. We analyzed habitat suitability and connectivity for the African elephant (*Loxodonta africana*) across PAs in south-western Tanzania in 2000, 2010, and 2019. We quantified land-use changes through remote sensing data; estimated habitat suitability through aerial survey data, remotely sensed variables and ensemble species distribution models; modelled least-cost corridors; identified the relative importance of each corridor for the connectivity of the PA network and potential bottlenecks over time through circuit theory; and validated corridors through local ecological knowledge and ground wildlife surveys. From 2000 to 2019, cropland increased from 7% to 13% in the region, with an average expansion of 634 km^2^ per year. Distance from cropland influenced elephant distribution models the most. Despite cropland expansion, the locations of the modelled elephant corridors (n = 10) remained similar throughout the survey period. Based on local ecological knowledge, nine of the modelled corridors were active, whereas one modelled corridor had been inactive since the 1970s. Based on circuit theory, we prioritize three corridors for PA connectivity. Key indicators of corridor quality varied over time, whereas elephant movement through some corridors appears to have become costlier over time. Our results suggest that, over the past two decades, functional connectivity across the surveyed landscape has largely persisted. Beyond providing crucial information for spatial prioritization of conservation actions, our approach highlights the importance of modeling functional connectivity over time and verifying corridor models with ground-truthed data.

## 1 Introduction

Habitat loss and habitat fragmentation are among the most serious threats to biodiversity conservation worldwide [1,2]. To counteract these trends, protected areas (PAs) are key conservation instruments [3–6]. However, accelerating human pressures in unprotected land adjacent to PAs, mainly through cropland and settlement expansion, increasingly isolate terrestrial PAs in many parts of the world [7–9], including savanna ecosystems of East Africa [10–12].

An effective way to ensure that PAs can meet their core conservation goals is to connect established PAs through corridors [13–15]. Well-designed and sufficiently protected wildlife corridors (defined here as a swath of land intended to allow passage by a focal species between two or more PAs [16]) facilitate animal movement between two PAs or across an entire PA network. From a biological perspective, such functional connectivity provides multiple benefits to wildlife populations. Corridors facilitate genetic exchange between sub-populations and thus support genetic diversity, enable species to track seasonal changes in food resources, allow for distribution shifts if the habitat of one area becomes unsuitable, for example due to climate change, enable natural recolonization in areas where a species went locally extinct, and expand the area and diversity of habitats beyond the boundaries of the PAs [17–19].

Functional connectivity between PAs is particularly important for large-bodied and wide-ranging terrestrial mammals, such as African savanna elephants (*Loxodonta africana*, hereafter elephants) [20–22]. Elephants have large home ranges [23,24], and they have shown remarkable site fidelity to their home ranges and movement routes even over multiple generations [25,26]. In Miombo ecosystems of Tanzania, elephant distribution also overlaps with the distribution of many other mammal species, suggesting that conserving corridors designed for elephants could also be beneficial for many other mammal species [19,27].

For centuries, elephant populations in East Africa have experienced multiple waves of human-caused mortality, primarily driven by the demand for ivory, interspersed with periods of population recovery [28–31]. In sum, elephant populations in East Africa are nowadays much smaller compared to historic baselines [31,32]. South-western Tanzania—the focus of this study—contains one of the few remaining elephant strongholds in Tanzania [31,33], yet recent surveys suggest that their populations are declining: in the Ruaha-Rungwa ecosystem, elephant numbers declined from 31,625 in 2009 to 20,090 in 2013 [34]; in the Katavi-Rukwa ecosystem the population dropped from 6,396 in 2009 to 5,738 in 2014 [35], and in the Ugalla ecosystem, their abundance declined from 4,000 in 2006 to 1,000 in 2009 [36]. While poaching is an immediate threat to the viability of elephant populations across the African continent [37–39], rapid, extensive and unplanned expansion of human land-uses in many parts of East Africa reduces the functional connectivity between PAs [21,38,40,41] and poses a threat for the long-term persistence of elephant populations in the region [22]. Recent genetic research shows incipient signs of genetic differentiation among elephant populations in south-western Tanzania [42,43], which indicates a potential lack of exchange between populations.

Although the locations of most wildlife corridors in Tanzania are broadly known (e.g. [17,41]), their exact locations and their current status are often obscured [44]. Ideally, the locations of wildlife corridors are informed by the actual movement of the target species [45,46], yet such data are rarely available for an entire PA network and over long time periods. As an alternative to animal movement data, presence data from periodically carried out aerial surveys provide a useful proxy for space use across large spatial scales [47]. Based on such presence data, species distribution models can be developed for target species [48,49] and the inverse of the habitat suitability (i.e. landscape resistance) can be used as input for modelling movement corridors across the PA network [50,51].

While corridor models based on available species distributions and remotely sensed data are routinely performed for large-scale conservation planning (e.g. [51]), we here aim to conduct a thorough assessment of the PA network connectivity in south-western Tanzania by adding four key elements. First, we include connectivity analyses over time (three snapshots during two decades) to identify the role of temporal processes affecting habitat suitability and connectivity [52–54]. Second, we include empirical data (i.e. elephant presence data) instead of solely relying on expert opinion (e.g. [51,55]) and parameterize species distribution models with natural landscape features (land cover, vegetation quality, terrain) and anthropogenic features (distance to cropland, distance to houses, distance to roads) instead of using land cover only (e.g. [51]) as input for modelling corridors. Third, we assess the relative importance of individual corridors to provide information for prioritizing conservation efforts on the ground. Fourth, we verify our corridor models through comparison with independent data [56]. In the absence of actual animal movement data [45], we utilize local ecological knowledge data obtained via interviews with key informants. Previous research suggests that local ecological knowledge can provide a robust validation dataset for habitat [57] and corridor use of large mammals [41,55,58].

To address our overarching goal of providing relevant information for the conservation of functional habitat connectivity across south-western Tanzania, we aimed at: (i) quantifying land-use changes in the region; (ii) modelling region-wide habitat suitability for elephants over time using ensemble distribution models; (iii) identifying least-cost corridors for elephant movement between PAs over time; (iv) identifying the relative importance of each modelled wildlife corridor; (v) identifying areas where elephant movement is constrained; and vi) validating the connectivity models by assessing whether identified corridors are reportedly used by elephants. We hypothesize that land-use conversion to agriculture has increased in southwestern Tanzania, and this in turn has resulted in a decline in the total amount of habitat suitability for elephants and a subsequent reduction in viable elephant corridors across the landscape.

## 2 Methods

### 2.1 Study area

**Fig 1:**
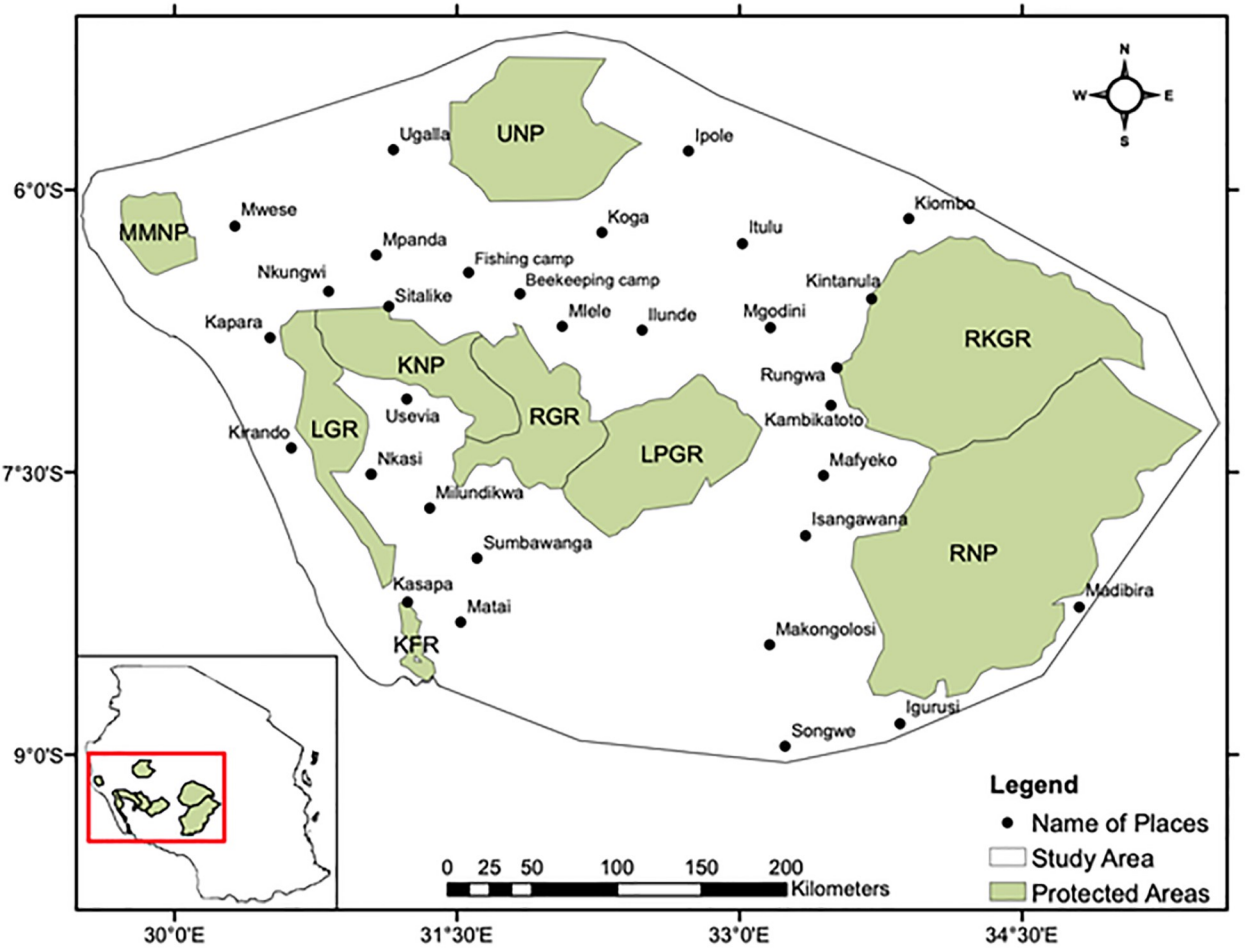
Map of the protected area network of south-western Tanzania, highlighting the spatial distribution of elephant’s core areas (KNP = Katavi National Park, RGR = Rukwa Game Reserve, LPGR = Lukwati-Piti Game Reserve, LGR = Lwafi Game Reserve, KFR = Kalambo Forest Reserve, MMNP = Mount Mahale National Park, UNP = Ugalla National Park, RKGR = Rungwa-Kisigo Game Reserve, RNP = Ruaha National Park) and major towns and interview sites/villages. The inset in the lower left shows the location of the study area within Tanzania.

Our study focused on south-western Tanzania (between 6° to 9° S and 30° to 35° E), which covers an area of about 187,308 km^2^ (Fig 1). The region is characterized by a mosaic of unprotected land (i.e. land that does not belong to a formal conservation category), and formally protected areas. Protection categories range from areas with little enforcement of human land-use restrictions (Game Controlled Areas: here, settlement, agriculture, livestock keeping are not allowed, but hunting on permit in specific hunting blocks is allowed), areas that allow regulated resource extractions such as Forest Reserves (here, limited timber and non-timber products extraction is permitted) and Game Reserves (here, touristic game hunting on permit is allowed) to strictly protected National Parks where human activities are restricted to photographic tourism and research [59,60]. Notable PAs in the study area include: Katavi National Park (KNP), Mahale Mountains National Park (MMNP), Ugalla National Park (UNP), and Ruaha National Park (RNP); Rukwa Game Reserve (RGR), Lukwati-Piti Game Reserve (LPGR), Rungwa-Kisigo Game Reserve (RKGR) and Lwafi Game Reserve (LGR); and Kalambo Forest Reserve (KFR) (Fig 1). The dominant large (> 90 kg) terrestrial mammal wildlife species include buffalo *Syncerus caffer*, elephant *Loxodonta africana*, eland *Taurotragus oryx*, giraffe *Giraffa camelopardalis*, hartebeest *Alcelaphus buselaphus*, greater kudu *Tragelaphus strepsiceros*, topi *Damaliscus lunatus*, roan antelope *Hippotragus equinus*, and zebra *Equus quagga* [35,60,61].

From 2000 to 2019 (the time period of our study), the area received between 350–2000 mm of annual rainfall, while the average annual temperature ranged between 13–28°C [62]. Elevation ranges from 600–2600 m asl. The vegetation mostly consists of miombo woodland, interspersed with *Acacia (Vachellia)-Combretum-Commiphora* woodland, grassland and flood plains [63]. Miombo woodland typically forms a single storey canopy of deciduous trees dominated by species of the genera *Brachystegia*, *Julbernadia*, and *Isoberlinia* [64]. From 2000 to 2012, the human population in rural Tanzania has grown from 26,025,846 to 38,691,642 [65]. In the study area, human population growth is partially fuelled by immigration from other regions [66]. The main land-use activities include agriculture and livestock keeping [66].

### 2.2 Elephant presence data and landscape variables

As a proxy for elephant space use across the study area, we used elephant presence data from periodically carried-out aerial surveys [47]. The main rationale was that such data were readily available at large temporal (three time steps during two decades) and spatial scales [32,36], and that aerial surveys relatively reliably detect elephants [67]. We are aware that corridors are ideally informed by movement data from collared elephants [68,69], yet such data were not available for our study area.

We obtained 339 (year 2000), 295 (year 2011), and 293 (year 2019) geo-referenced detections of elephant groups from the Tanzania Wildlife Research Institute (TAWIRI). Aerial surveys were conducted during the dry season following the systematic reconnaissance flight technique as described by [70]. While elephants often range more widely during the wet season [71], wet season surveys for the Katavi region are scarce [72] and likely fail to detect a substantial proportion of elephants due to limited visibility caused by green crown cover [73]. Based on established relationships between the distribution of large savanna mammals in Tanzanian ecosystems and landscape features [55,74,75], we selected the following landscape variables in our habitat suitability model: land cover (as a proxy for habitat structure and land-use), Enhanced Vegetation Index (EVI; as a proxy for primary productivity due to its advantages of reducing the background noise, atmospheric noise, and pixel saturation in most cases compared to NDVI during the dry season [76]), elevation, slope, topographic wetness index, terrain ruggedness and proximity to cropland, roads, houses, rivers, and rainfall. We obtained the global 30 m SRTM digital elevation model (DEM) for the study area from the U.S. Geological Survey (https://earthexplorer.usgs.gov) and used the DEM to derive slope, topographic wetness index and terrain ruggedness index using QGIS 3.16 [77]. We obtained spatial layers for houses for the years 2000 and 2011 from TAWIRI [61] and for the year 2019 from OpenStreetMap (http://download.geofabrik.de/africa/tanzania.html). We obtained spatial layers for roads and rivers for a single time step (i.e., 2019) from OpenStreetMap, under the assumption that these features did not change substantially across our study period. For all three spatial layers (houses, roads and rivers), we generated distance raster surfaces at a resolution of 30 m using the Euclidian distance tool in ArcMap 10.6 [78]. We obtained the annual rainfall at a resolution of 5 km for each year for the study area from CHIRPS (https://data.chc.ucsb.edu/products/CHIRPS-2.0/). We generated EVI raster surfaces for the dry season (i.e. between July and September) of each year from Google Earth Engine—Landsat 5/8 Collection 1 Tier 1 8-Day EVI Composite [79]. We projected all layers to the same projection and resampled them to 1 km resolution.

### 2.3 Spatial distribution of cropland

To produce land cover maps as inputs for land-use change analyses, habitat suitability and connectivity modelling, we acquired readily available 30 m resolution Landsat 5 and Landsat 8 imagery from U.S. Geological Survey’s Earth Explorer (https://earthexplorer.usgs.gov/) for each time step. Our choice of date for satellite imagery was based on availability of aerial survey data for the dry seasons between 2000 and 2019, and imagery free from cloud cover. We used the atmospheric correction algorithm ATCOR to remove haze and calculated the top of atmosphere reflectance for Landsat 5 and Landsat 8 imagery using PCI Geomatica version 2018 [80]. For land-use classification, we generated 600 training polygons for each year through high-resolution Google Earth images and field knowledge [75,81]. We used the scatterplot tool to evaluate our training samples to assess if there was enough separation between land cover classes using ArcMap [78]. Subsequently, we employed a supervised classification approach using a support vector machine algorithm to classify satellite imagery [82,83], which allowed us to condense land cover to five major categories: dense woodland, open woodland (to create a few land cover classes that are more representative of the entire study area, grasslands and shrublands were combined with the open woodland class), burned areas (including burned areas of grasslands, shrublands, open woodland, and closed woodland), cropland, and water bodies. To assess the accuracy of our classified maps, we generated a total 1800 (i.e., 600 points per time step) accuracy assessments points using stratified random sampling in ArcMap. We used high-resolution images from Google Earth and base-map layers from Google Satellite, ESRI Satellite, and Bing Satellite available in ArcMap and QGIS to validate our land cover maps [84–86]. Our overall land cover classification accuracy for the three years ranged from 96% to 98% with kappa coefficients ranging between 0.95 and 0.97 (S1 Table).

### 2.4 Modeling habitat suitability

To avoid potential problems arising from collinearity, we tested variables for cross correlations using the corrplot package [87], and selected only variables with Pearson’s correlation coefficient (r) ≤ 0.7 [88]. Due to a strong correlation between ‘slope’ and ‘terrain ruggedness index’, we removed the variable ‘terrain ruggedness index’, and used ten uncorrelated variables to fit elephant distribution models for each study period. We used elephant presence data with a background mask to generate 1000 pseudo-absences as response variable and environmental data as explanatory variables to build an ensemble model for each study period using the SDM package [89]. Ensemble modelling uses multiple modelling algorithms, a strategy that minimises uncertainty associated with a single modelling approach and increases the accuracy of model predictions [90]. The ensemble model included the following algorithms: maximum entropy (Maxent), generalized boosted model (GBM), generalized additive model (GAM), and random forest (RF). We selected these algorithms based on their predictive power (high AUC values) obtained from the model run. For each algorithm, we ran 10 replications in which 75% of the presence points were used to train the model and the remaining 25% were used to test the model [91]. We used the area under curve (AUC) of the receiver operating characteristic (ROC) to evaluate the accuracy of four distribution models [92,93]. To build the ensemble model, we used a weighted-averaging approach whereby individual models were weighted according to their predictive accuracy [89,94]. We used the AUC > 0.85 of the ROC to evaluate the performance of the ensemble model [89,95,96]. Based on the output obtained from the models for the three study periods, we predicted habitat suitability for the entire landscape for each of the three years. To visualize the spatial and temporal dynamics of suitability maps for elephants over time, we categorized habitat suitability into three classes (high, moderate, and marginal) according to the natural breaks classification technique [97,98]. We used the Jack-knife test to assess the relative contribution of each predictor in the final habitat suitability model for each study period.

### 2.5 Modeling habitat connectivity

For each time step, we modelled the connectivity across the PA network using Linkage Mapper [99]. As input data, we considered the polygon feature class containing core areas as source locations and a resistance surface map. As source locations we used the PAs (including areas annexed in 2006) that are known to be occupied by elephants (i.e., all PAs that are displayed in Fig 1). To estimate landscape resistance, we transformed the habitat suitability values into resistance values using the approximately linear (i.e., the factor c = 0.25) transformation function [50]: *R* = 100–99 * (((1- exp(-*c* * *h*))/(1 –exp(-*c*))), where *R* is resistance, *h* is suitability, and the factor *c* determines the shape of the curves. For this transformation, resistance equals 1 when habitat suitability is 1 and resistance equals 100 when habitat suitability is 0. Beforehand we tested four different values for the shape factor *c*: 2, 1, 0.5, and 0.25; and the 0.25 value was chosen for further analysis because it resulted in reasonable corridor widths without the loss of multiple corridors connecting PAs in our study area. We used the linkage mapper to create least-cost corridors between PAs based on calculated cost-weighted distance (CWD) [99]. Due to a lack of empirical data on the optimum width of CWD for African savanna elephants, we tested four different maximum cost-distance values: 200, 150, 100, and 50 cost-weighted kilometers (cw-km). We chose the 200 cw-km cutoff value (i.e. the largest default threshold in Linkage Mapper Connectivity Analysis Software [99]) for further analysis because it resulted in reasonable corridor widths without the loss of multiple corridors connecting PAs, and appeared wide enough to facilitate movement. Then, we truncated the least-cost corridors at 200 cw-km and this threshold was used to clip the least-cost corridors across the study period. To quantify the characteristics of each resulting least-cost corridor we used Centrality Mapper (which calculates the sum of all current density values) and Pinchpoint Mapper (which generates current-maps that identify and map pinch-points i.e., constrictions or bottlenecks) that utilize circuit theory [100], and treat resistance surface as the hindrance between PAs [50]. We used the Centrality mapper [101] to identify the corridors most important for maintaining the connections among the networks (i.e., gatekeepers of connectivity), and Pinchpoint Mapper [102] to identify bottlenecks (i.e., locations of the corridors where animal movement is restricted due to unfavorable landscape and anthropogenic features). We used two metrics to describe the quality of each corridor [49,103]. First, the ratio of CWD to the Euclidean distance (EUD) separating each pair of PAs; this value indicates how difficult it is to move between PAs relative to how adjacent they are. Second, the ratio of CWD to the length of least critical path (LCP); this value indicates the average resistance along the optimal path between the PAs. For both metrics, high quality corridors are characterized by a ratio close to 1 [104].

### 2.6 Validating habitat and corridor models

To validate our habitat suitability maps, we collected evidence for elephant presence (i.e., direct observations, elephant dung) along 105 three-kilometres transects within the Katavi-Rukwa ecosystem. We chose this subset of the study area for our validation approach as it is centrally located within the study region and allowed us to sample across a wide range of PA categories. We divided our validation study area into a 5 km by 5 km grid, so that transects were separated by 5 km to minimize spatial autocorrelation of our independent validation data. We surveyed each transect once during the dry season between July and September 2020. In each grid, we recorded centroid coordinates, and along each transect, we recorded the presence/absence of elephant dung. To validate our predictive maps, we first condensed our data to presence-absence; in 63 of the 105 transects we detected elephant dung; in the remaining 42 transects, we did not detect elephant dung. Second, we applied the specificity-sensitivity threshold [105,106] to convert our continuous suitability maps into binary maps (i.e., suitable and marginally suitable areas). Third, we superimposed presence data on the current 2019 binary map and used the extraction tool to extract the binary values to elephant presence data for accuracy assessment using ArcMap (S1 Fig).

To validate our least-cost corridors, we conducted key informant interviews in sites (i.e., beekeeping and fishing camps) and villages close to the least-cost corridors generated within our study area. For each modelled corridor between two PAs, we conducted interviews at 1 to 3 villages or fishing/beekeeping camps: KNP-UNP (Uruwira fishing camp) and RGR-UNP Mlele (beekeeping camp), LPGR-UNP (Ilude-Koga), UNP-RHGR (Ipole, Itulu, Mgodini), LPGR-RKGR (Kambikatoto), LPGR-RNP (Isangawana), LGR-MMNP (Kapara, Nkungwi), LGR-KFR (Kasapa), MMNP-UNP (Ugala, Mwese), and RKR-KFR (Milundkikwa) (see Fig 1 for the locations of the villages and camps). At each fishing/beekeeping camp, we interviewed 5 persons. In each village, we interviewed 10 persons: one village executive officer and/ or village chairperson, one beekeeper or fisherman, four members of natural resource committees or village game scouts, and four farmers and/ or pastoralists using semi-structured interviews. Prior to the fieldwork, we reported to the village office and asked for permission to conduct the interviews. We also involved the village leaders in selecting suitable interviewees (i.e. people with extensive wildlife knowledge; above the age of 18 years; and resident of the area since 2000). When approaching interviewee candidates, we explained the purpose of the study, promised that their identities would remain anonymous, and asked for consent to participate in an interview. The key interview questions were: i) “How many individual elephants have you seen in this area during the last year and where was this?”, and ii) “What season of the year did you see the elephants?”.

### 2.7 Ethics statement

All research was carried out with permission from the Tanzanian Wildlife Research Institute (TAWIRI) and the Tanzanian Commission for Science and Technology (COSTECH), permit #: 2021-372-NA-2021-77. A respondents’ verbal consent was obtained before starting an interview because the majority of them are not competent in writing as advised by village leaders. No names were gathered, and respondents’ identities were numerically coded to maintain anonymity.

## 3 Results

### 3.1 Cropland cover and habitat suitability for elephants

**Fig 2:**
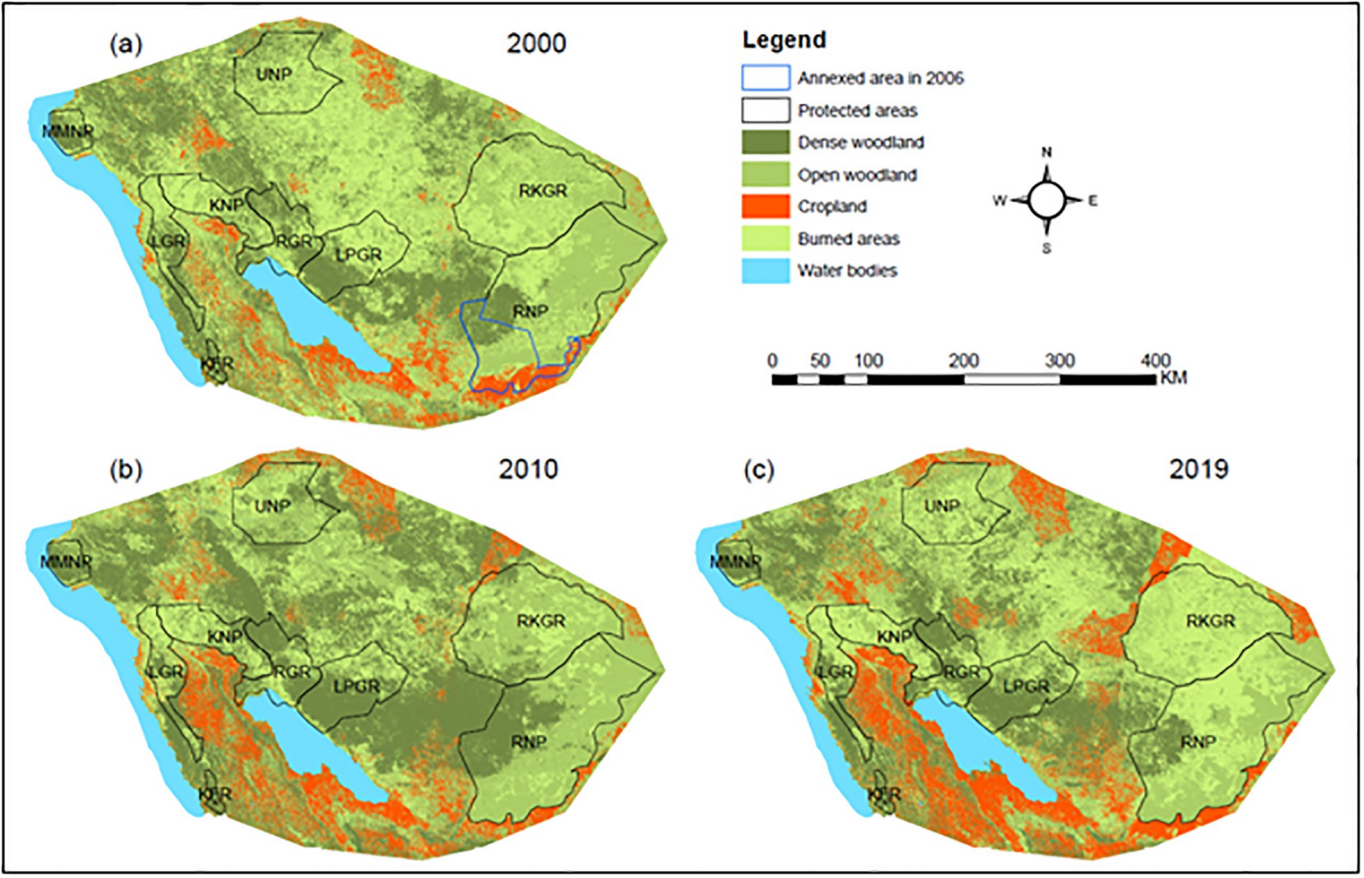
Map of the study region, showing the estimated distribution of cropland in (a) 2000, (b) 2010, and (c) 2019. The polygon with the blue colour in (a) 2000 indicates area annexed by the RNP in 2006.

From 2000 to 2019, the study area has experienced substantial land-use changes (Table 1). Most notable is a substantial increase of cropland cover from 6.71% (12,568 km^2^) in 2000 to 13.14% (24,612 km^2^) in 2019 (Fig 2 and Table 1). On average, a total of 0.34% of surface area was converted to cropland every year (634 km^2^/year). By 2019, cropland cover approached the borders of all PAs in south-western Tanzania (Fig 2).

**Table 1:** Extent and rate of land cover changes between 2000 and 2019 in south-western Tanzania (Area covered = 187,308 km^2^).

Land cover type	Area (%)			Change in land cover (%)			Average annual rate of change (2000–2019)	
	2000	2011	2019	2000–2011	2011–2019	2000–2019	(%)	(Km^2^)
Dense woodland	22.38	33.38	23.77	10.99	-9.61	1.39	0.07	136.57
Open woodland	38.43	38.45	31.17	0.03	-7.29	-7.26	-0.38	-715.36
Cropland	6.71	8.79	13.14	2.08	4.34	6.43	0.34	633.55
Burned areas	24.70	11.70	24.41	-13.00	12.71	-0.29	-0.02	-28.52
Water bodies	7.78	7.67	7.51	-0.11	-0.16	-0.27	-0.01	-26.31

**Fig 3:**
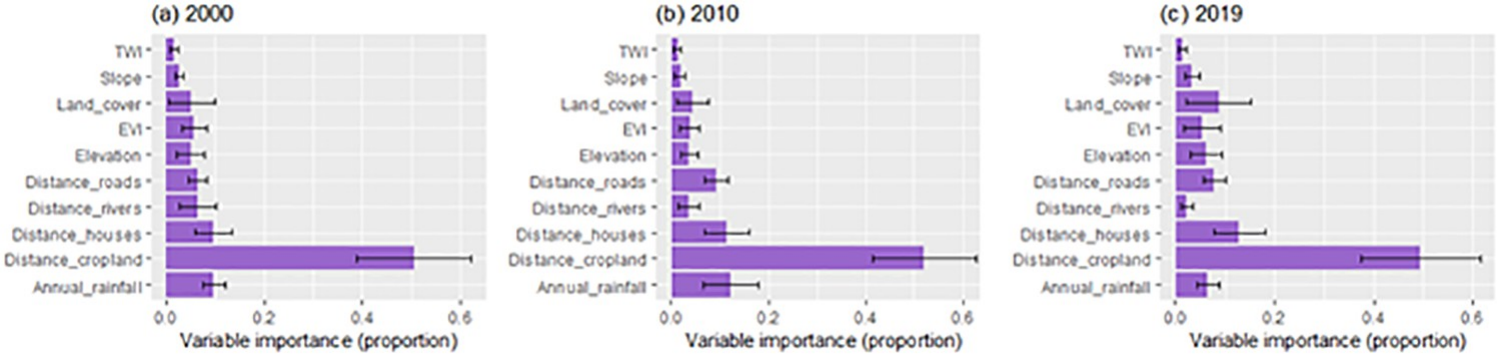
Relative contribution of predictor variables for predicting the potential habitat distribution of elephants in south-western Tanzania.

Our model evaluation results suggested that all ensemble habitat suitability models for each time step had good performance, with weighted average AUC scores above 0.8 (Table 2). Among the variables influencing survey-specific habitat suitability for elephants, distance to cropland consistently contributed most (Fig 3). Based on the validation data collected in 2020, our overall predictive map accuracy for 2019 was 92.06%.

**Table 2:** Accuracy evaluation of the habitat suitability for elephants between 2000 and 2019 (AUC: Area under the curve of the receiver-operating characteristic).

Methods	AUC		
	2000	2010	2019
Maximum entropy algorithm (Maxent)	0.85	0.86	0.85
Generalized boosted models (GBM)	0.85	0.85	0.84
Generalized additive models (GAM)	0.84	0.85	0.86
Random forest models (RF)	0.95	0.94	0.94
**Weighted average**	**0.87**	**0.88**	**0.87**

**Fig 4:**
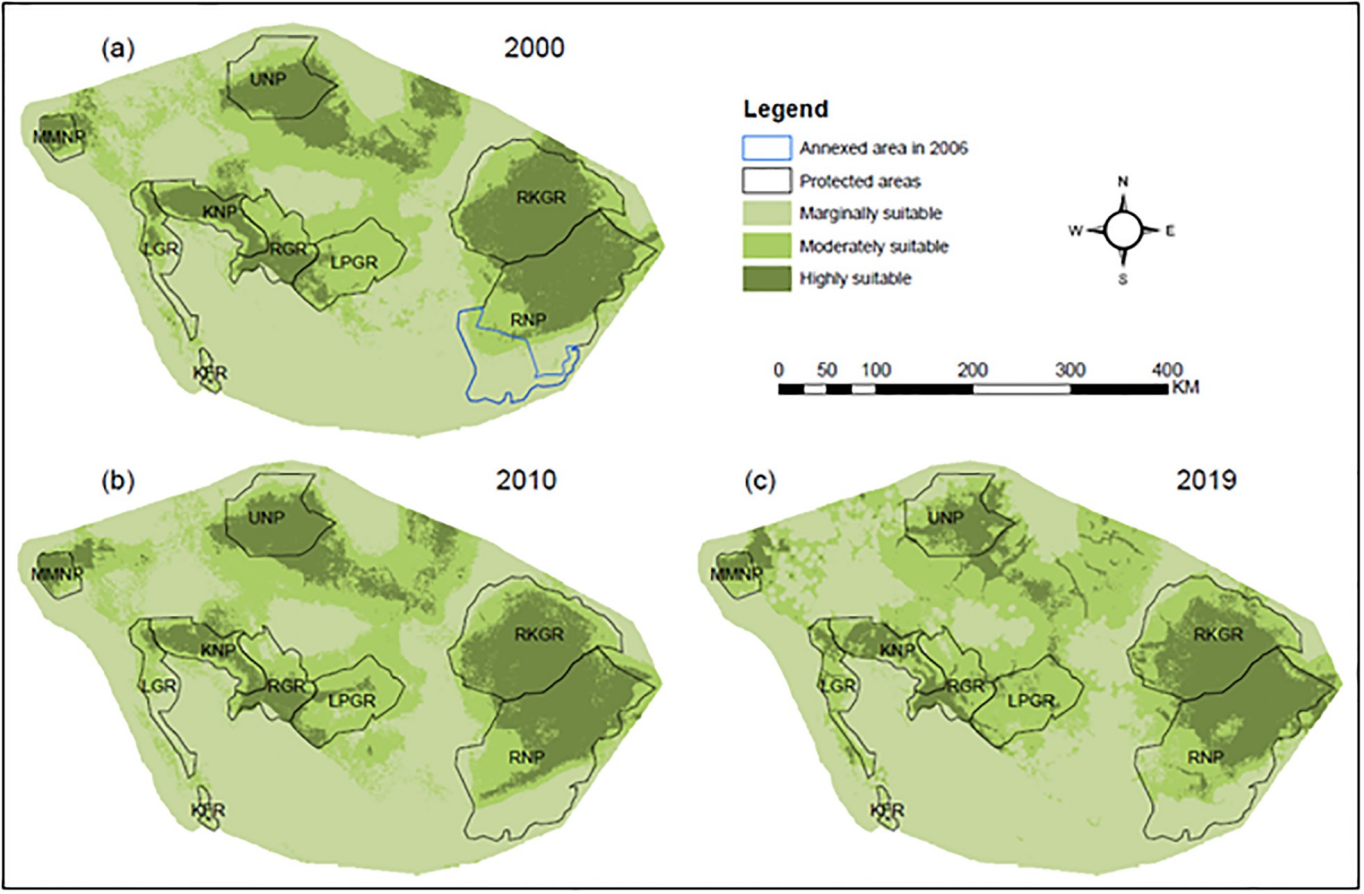
Map of habitat suitability, indicating spatial and temporal distribution of potential habitat for elephant within protected areas network of south-western Tanzania. The polygon with the blue colour in (a) 2000 indicates area annexed by the RNP in 2006.

Predictions of our ensemble model suggest that highly suitable elephant habitat declined over time: in 2000, 21.11% of the area was highly suitable for elephants, in 2011, this area was reduced to 20.25%, and in 2019, it was further reduced to 17.32% of the surveyed region (Fig 4). At each time step, large portions of highly suitable habitat fell within the boundaries of PAs (2000: 16.57%; 2011: 16.28%; 2019: 14.14%; Fig 4). Small pockets of highly suitable habitat were widely distributed in the eastern part of MMNP, the southern part of UNP, and the northern part of RKGR. Across the study period, most areas outside the PAs were classified as marginally suitable elephant habitat.

### 3.2 Connectivity models for elephants

**Fig 5:**
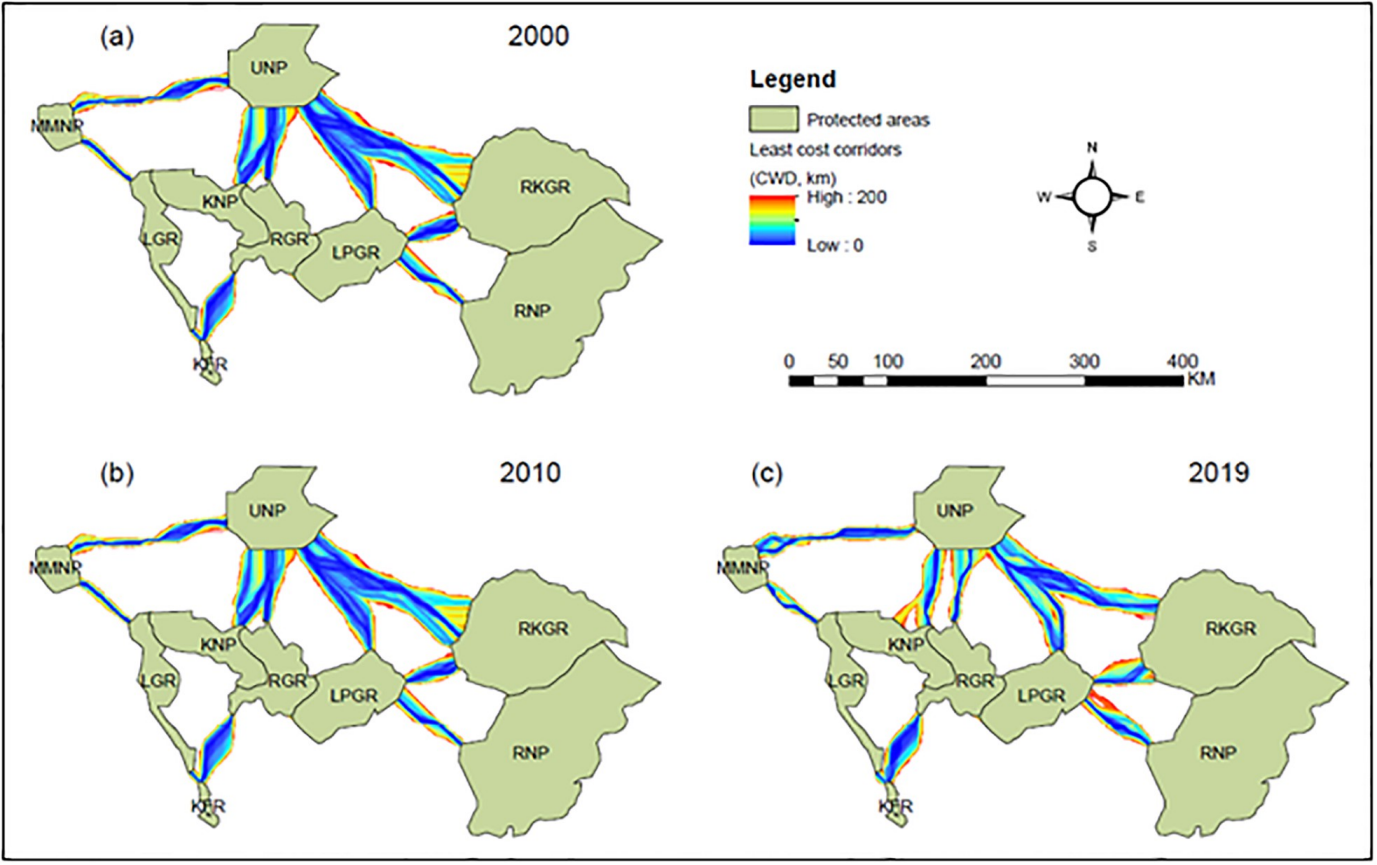
Map of least-cost corridors clipped at cost-weighted distance (CWD) of 200 km, depicting spatial and temporal distribution of elephant corridors.

For each time step, our modelling approach identified ten elephant corridors across the PA network (Fig 5 and Table 3). The average resistance encountered by elephants along an optimal path between corridors varied over time (Table 3). Highest CWD:EucD ratios were recorded for the corridors linking RGR-KFR, LGR-KFR, and LPGR-RNP indicating that the cost of elephant movement between these PAs was higher than for other pairs of PAs throughout the study period (Table 3). The corridors between LPGR-UNP and RGR-UNP exhibited the lowest CWD:EucD ratios, suggesting that the costs for elephants to move between these PAs were lower than for other pairs of PAs throughout the study period (Table 3). Similarly, the highest CWD:LCP ratios were recorded for the corridors between RGR-KFR, LGR-KFR, and LPGR-RNP, indicating that the average resistance encountered by elephants along the optimal path between PAs was higher than for other corridors throughout the study period (Table 3). The lowest CWD:LCP ratio was estimated for the corridors between LPGR-UNP and RGR-UNP, indicating low resistance for elephant movement along these corridors (Table 3).

**Table 3:** Attributes of 10 corridors mapped between PAs in south-western Tanzania. Corridors are sorted by decreasing centrality scores to demonstrate their importance in keeping the protected areas network connected.

PA		^a^CWD:EUCD			^b^CWD:LCP			Current flow centrality (Amps)			Sightings	^c^Sightings	Interview partners	Corridor
From	To	2000	2010	2019	2000	2010	2019	2000	2010	2019	Mean ± SD	Season	N	status
LPGR	RKGR	84.90	85.54	89.86	77.51	78.09	82.03	7.34	7.31	7.09	7.80 ± 2.20	Wet and Dry	10	Active
LGR	KFR	96.05	89.21	91.25	88.88	77.43	82.18	6.91	6.90	6.93	21.90 ± 3.81	Wet	10	Active
LGR	MMNP	82.69	71.51	66.88	78.03	67.48	59.37	6.37	6.62	6.78	8.40 ± 3.44	Wet and Dry	20	Active
LPGR	RNP	86.65	86.27	86.07	81.54	81.79	81.00	4.79	4.82	4.92	10.40 ± 3.80	Wet and Dry	10	Active
RGR	UNP	64.48	63.85	68.40	61.13	56.63	56.25	3.62	3.54	3.60	18.40 ± 1.67	Wet	5	Active
UNP	RKGR	71.44	72.97	69.85	58.77	63.22	62.41	3.44	3.36	3.33	10.00 ± 3.15	Wet	30	Active
MMNP	UNP	71.21	71.77	72.26	64.09	65.16	65.74	3.28	3.05	3.00	11.50 ± 3.05	Wet and Dry	20	Active
KNP	UNP	72.23	68.58	70.67	62.79	63.06	64.70	3.03	3.09	3.09	16.60 ± 2.15	Wet	5	Active
LPGR	UNP	60.16	61.98	61.48	56.21	54.66	53.36	2.25	2.11	2.19	13.70 ± 1.88	Wet and Dry	10	Active
RGR	KFR	98.04	90.97	96.11	89.70	83.23	87.93	1.25	1.23	1.21	0	-	10	Inactive

^a^Index describes the cost of elephant movement between PAs relative to how adjacent they are.

^b^Index represents the average resistance encountered by elephants along an optimal path between PAs.

^c^Wet season (runs from the end of October right through to December, and then again from the end of March to the beginning of June), and dry season (runs from end of June to the beginning of October).

### 3.3 Relative corridor importance and bottlenecks

**Fig 6:**
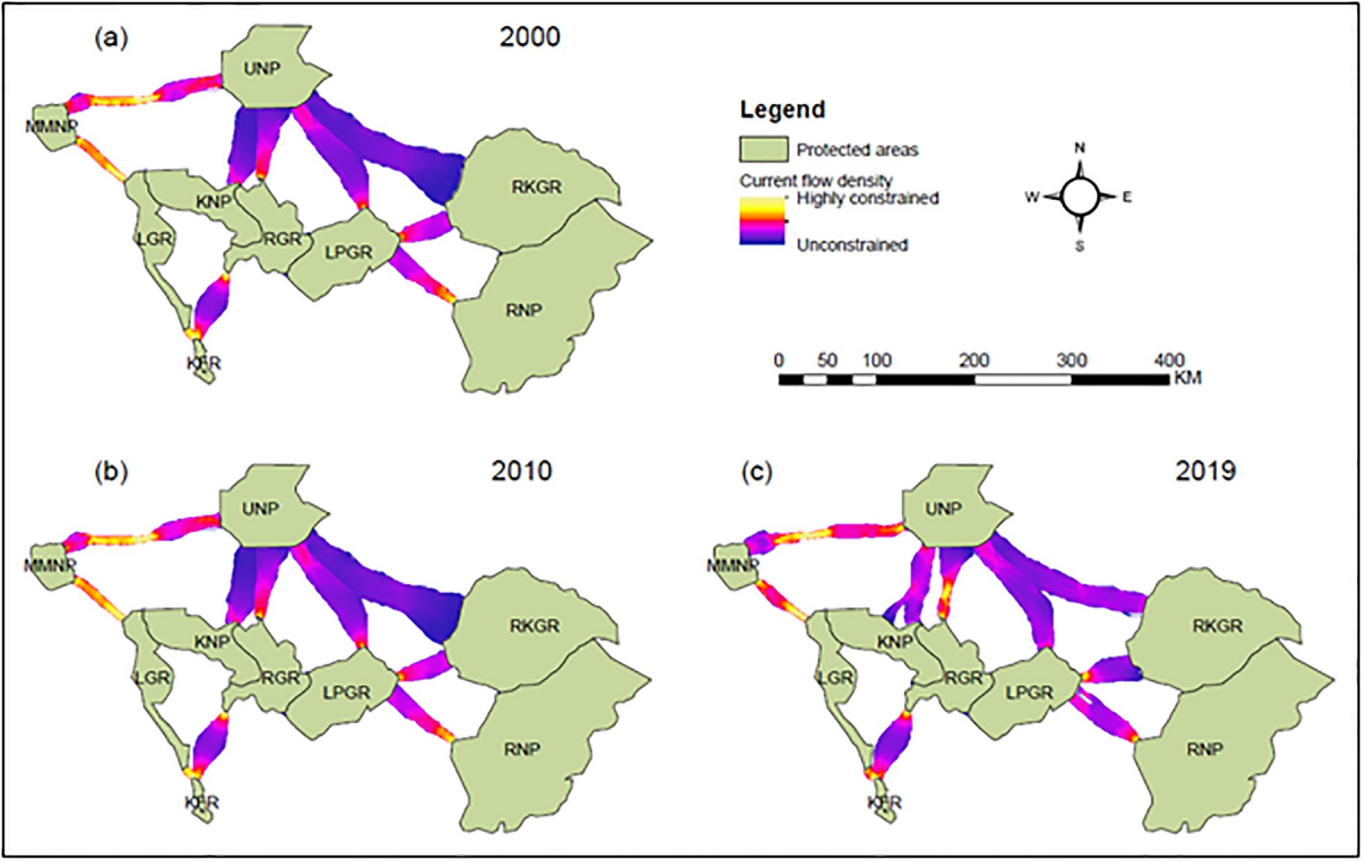
Pairwise pinch-point maps indicating where the current flow is highly restricted between the two protected areas. Yellow shades indicate areas where the current flow is highly restricted representing the pinch-points (i.e., constrictions or bottlenecks) due to natural and human-made landscape features.

Across time, the main corridor locations remained relatively constant (Figs 5 and 6). However, our network link centrality analyses showed that the corridors between LPGR-RNP, LGR-KFR, and LGR-MMNP recorded the highest centrality scores throughout the study period, highlighting their importance for overall connectivity in the region (Table 3). For all time steps, the lowest centrality score was recorded for the corridor between RGR and KFR, indicating its apparent minor role for overall connectivity (Table 3). Our pinch-point analyses also showed that areas with high current flow density represented corridor bottlenecks (Fig 6). Despite the spatial location of bottlenecks remaining relatively consistent across time, its current flow density increased across corridors over time (S2 Table).

The corridors between MMNP-UNP, LGR-MMNP, and LGR-KFR, exhibited the highest current flow density, suggesting that elephant movement is more restricted at the corridor margins by physical barriers such as anthropogenic and topographical features than for other pairs of PAs throughout the study period (Fig 6, S2 Table). The corridors between UNP-RKGR, KNP-UNP, and LPGR-UNP recorded the lowest current flow density, highlighting that elephant movements are less restricted on the corridor margins by physical barriers throughout the study period (Fig 6).

### 3.4 Validating elephant corridors

To validate our corridors, we systematically gathered local ecological knowledge from 130 interview partners in one fishing camp, one beekeeping camp and 12 villages in the vicinity of the modelled corridors. Out of 130 respondents, 120 (i.e. 92%) reported that they saw at least one elephant during the last year (Table 3). Out of the ten modelled corridors, respondents confirmed that nine were used by elephants in the year prior to the interviews (Table 3). In five of these locations, interview partners reported that they observed elephants during the wet (October-December; March-June) and dry season (end of June to the beginning of October; January-February) in the camp and village area (Table 3). In four locations, respondents reported elephant presence for the wet season only (Table 3). In the RGR-KFR corridor, none of the interview partners had seen elephants in the year prior to the interviews. According to the interview partners, elephants used this corridor until the 1970s. Among the active corridors, interview partners reported the highest relative numbers of elephants in areas located near corridors linking LGR-KFR, RGR-UNP, and KNP-UNP (Table 3).

## 4 Discussion

Our data-driven and field-validated models of elephant habitat and corridors over two decades suggest that functional connectivity for elephants is largely persisting in this region of south-western Tanzania. However, the loss of at least one elephant corridor and the observed rapid pace of land-use change calls for timely conservation action to protect and partly restore the functional connectivity in this landscape.

### 4.1 Drivers of habitat suitability and functional connectivity

Our results indicate that the habitat suitability for elephants in our study area is more determined by anthropogenic variables than by environmental variables. Among the variables influencing habitat suitability, distance to cropland and houses consistently contributed most (Fig 3). These findings echo results of other studies which have shown that elephants and other large East African mammals avoid cultivated and settled areas [75,107–109], highlighting that the expansion of human settlements and agriculture pushes and compresses the distribution of large wildlife species further into the core areas of PAs. This pattern has previously been shown for large herbivores in the Katavi-Rukwa ecosystem in western Tanzania [75] and the Serengeti ecosystem in northern Tanzania [110]. While an evaluation of connectivity typically relies on movement data, our results on habitat suitability and elephant corridors derived from two independent presence/absence datasets (dung survey and local ecological knowledge) mirror elephant-habitat relationships [e.g. avoidance of human influence (i.e., settlements and cropland)] that were found in elephant movement-habitat studies [24]. However, as our analyses were based on elephant space-use during the dry season, our models may not fully capture elephant movement. In some ecosystems of East Africa, elephant movements outside of PAs mostly occur during the rainy seasons [47,111] and thus dry season distributions may provide rather conservative models for landscape-scale distribution and functional connectivity. Nevertheless, interview-based data suggest that the modelled corridors [which were based on imperfect data–a common issue for documenting dynamic processes such as long-distance migrations [112]] are used by elephants during both dry and wet seasons (Table 3). Additional research could elucidate which areas of the ecosystem are primarily used for connectivity and which patches are additionally used as stop-over or longer-term habitat [19].

Anthropogenic change such as expansion of human settlements and agriculture towards PAs are often associated with habitat destruction, encroachment and blockage of wildlife corridors [24,113]. Among the ten key corridors identified, one was inactive and had been blocked by human settlements and agriculture before the start of our study. The remaining 9 active corridors were characterized by increasing movement costs over time and contraction caused by expansion of human settlements and agriculture (S2 Fig). Albeit still being in use, our land-use change analyses demonstrated that by 2019 most of our corridors (7 out of 10, i.e., RGR-KFR, LPFR-RKGR, LPGR-RNP, UNP-RKGR, MMNP-UNP, LGR-MMNP, and LGR-KFR) were encroached by cropland in the study region (S2c Fig). Between 2000 and 2019, cropland cover increased by 634 km^2^ per year, mostly at the expense of natural vegetation (i.e., dense and open woodlands) around PAs (Fig 2). This land-use change in the region is likely associated with an increase of the rural human population [65], which is partially elevated by immigration from other regions [66]. Agriculture (e.g., rice, maize, cotton, tobacco) and livestock keeping are the main land-use activities in the region [66]; the former is practiced in an unsustainable manner (i.e., shifting cultivation) particularly for tobacco production. Low fertility of the regions’ soils [114] and the nutrient-demanding nature of tobacco [115], as well as demand for biomass energy to dry the tobacco leaves [116] are the primary reasons for shifting cultivation in the region. Such shifting cultivation likely contributed to land-use change near elephant corridors and near core protected areas in the study region (S2a–S2c Fig). Importantly, the expansion of the RNP in 2006 resulted in further displacement of Usangu farmers and Sukuma pastoralists from Mbarali District to frontier areas around the western part of the park [117]. Likely, such migration to frontier areas around the western part of RNP contributed to the observed encroachment within LPGR-RNP and LPGR-RKGR corridors (S2a–S2c Fig). If the observed trend in land-use change continues, elephant movements within the study region will most likely be hampered further in the near future (e.g., through RGR-KFR, LPFR-RKGR, LPGR-RNP, UNP-RKGR, LGR-KFR, and MMNP-UNP corridors), with anticipated negative consequences on population viability of elephants.

### 4.2 Conservation implications

The study region is one of the few regions in Africa where large scale movements of elephants seem to persist [118,119], yet our results suggest that this functional connectivity is increasingly threatened by anthropogenic land conversion for human settlements and cropland. A recent study recorded evidence of genetic differentiation among the elephant populations from the Ruaha‐Rungwa and Katavi‐Rukwa ecosystems in the study region [42]. Although the levels of genetic differentiation recorded were low and mainly concerned the younger cohort, it still indicates a recent divergence likely caused by reduced habitat connectivity between the two ecosystems in the study region [42]. Linking PAs with corridors is a cost-effective way to safeguard functional connectivity within and across ecosystems and requires relatively little land as corridors do not necessarily need to be very wide. The importance of wildlife corridors is also reflected by the Wildlife Corridor Act of the Tanzanian government [120,121]. Based on our analyses, we recommend the following conservation actions to retain, restore and possibly even enhance ecological connectivity in the study region. First, in areas where corridors get narrower (e.g., MMNP-UNP, LGR-MMNP, and LGR-KFR) and encroached (e.g., LPFR-RKGR, LPGR-RNP, UNP-RKGR, MMNP-UNP, and LGR-MMNP), delineation of the corridor would be a first crucial step in conserving wildlife corridors. This also requires enforcing land-use plans and having alternative and sustainable forms for generating income from the land without impairing wildlife habitat. One possible avenue for implementation would be generating a forest and wildlife-based economy from the modelled corridors (e.g., income from selling carbon credits, beekeeping, and ecotourism) in seeking to generate benefits from nature to outweigh the costs associated with wildlife conservation. For example, Carbon Tanzania established a REDD+ project that secures habitat in the corridor linking MMNP and KNP corridor through LGR and provides adjacent communities with income through the sale of carbon credits. Second, in highly degraded areas within some modelled corridors (e.g., RGR-KFR, LPGR-RKGR, MMNP-UNP) due to human encroachment from arable farming, restoration may be an option through natural regeneration of miombo from roots and cut stumps [122–124]. However, despite a high regeneration potential, long-term reforestation of native miombo species planning is required [125] to restore highly degraded areas. Third, our models provide spatially explicit locations of wildlife corridors. Thus any future development projects (e.g., upgrading earth roads to tarmac roads) and activities impairing wildlife habitat should be prioritized outside of the modelled corridors to avoid further impacts on connectivity. In sum, a long-term strategy would be to incorporate PA networks into land-use plans that integrate the needs of both people and wildlife [126,127]. Our modelled elephant corridors and land-use change maps for the two past decades could offer valuable inputs for such landscape planning.

## 5 Conclusions

Conserving functional connectivity is vital for the long-term persistence of wide-ranging mammals, such as elephants. Our findings on connectivity in the Miombo region of south-western Tanzania could help in the delineation, restoration, and conservation of elephant corridors. Elephant connectivity can be maintained or restored by reducing further anthropogenic cropland expansion towards the modelled corridors through implementation and enforcement of site-specific land-use planning. Our analysis integrates several temporal data sources (from remote sensing and aerial wildlife surveys), models (ensemble species distribution models, least-cost and circuit theory approach) and validation techniques (local ecological knowledge and ground wildlife surveys) to model spatially explicit wildlife corridors for effective PA network land-use planning and conservation. The approach can also be reproduced in other regions and for other wildlife species.

## Supporting information

S1 Fig Validation map, presenting presence and absence of elephants within our predicted binary maps (i.e., suitable and marginally suitable areas) in 2019. Each dot represents a three kilometer long transect.
(DOCX)

S2 Fig Map of the study region, showing the cropland distribution within and around the modelled corridors in (a) 2000, (b) 2010, and (c) 2019. The polygon with the blue colour in (a) 2000 indicates area annexed by the RNP in 2006.
(DOCX)

S1 Table Cross-tabulation error matrix for 2000, 2010 and 2019 land cover classification in south-western Tanzania. (DOCX)

S2 Table Distribution of current flow density (Amps/cell) within the modelled corridors between PAs across three time steps (2000, 2010, and 2019) in south-western Tanzania. (DOCX)
