# Supplementary material for: Modelling elephant corridors over two decades reveals opportunities for conserving connectivity across a large protected area network
Source: PLoS One. 2023 Oct 13;18(10):e0292918. doi: 10.1371/journal.pone.0292918 (PMC10575508; doi:10.1371/journal.pone.0292918)

S1 Fig. Validation map, presenting presence and absence of elephants within our predicted binary maps (i.e., suitable and marginally suitable areas) in 2019. Each dot represents a three kilometer long transect.


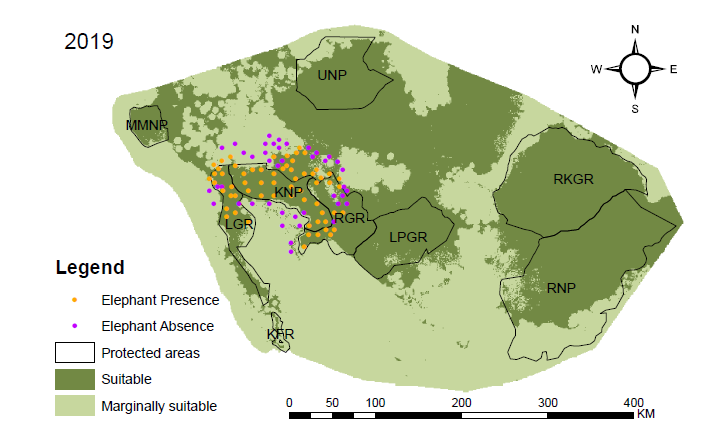

Supplement: S1 Fig — Each dot represents a three kilometer long transect. (DOCX) [file pone.0292918.s001.docx]
