# Supplementary material for: Modelling elephant corridors over two decades reveals opportunities for conserving connectivity across a large protected area network
Source: PLoS One. 2023 Oct 13;18(10):e0292918. doi: 10.1371/journal.pone.0292918 (PMC10575508; doi:10.1371/journal.pone.0292918)

S2 Fig. Map of the study region, showing the cropland distribution within and around the modelled corridors in (a) 2000, (b) 2010, and (c) 2019. The polygon with the blue colour in (a) 2000 indicates area annexed by the RNP in 2006.


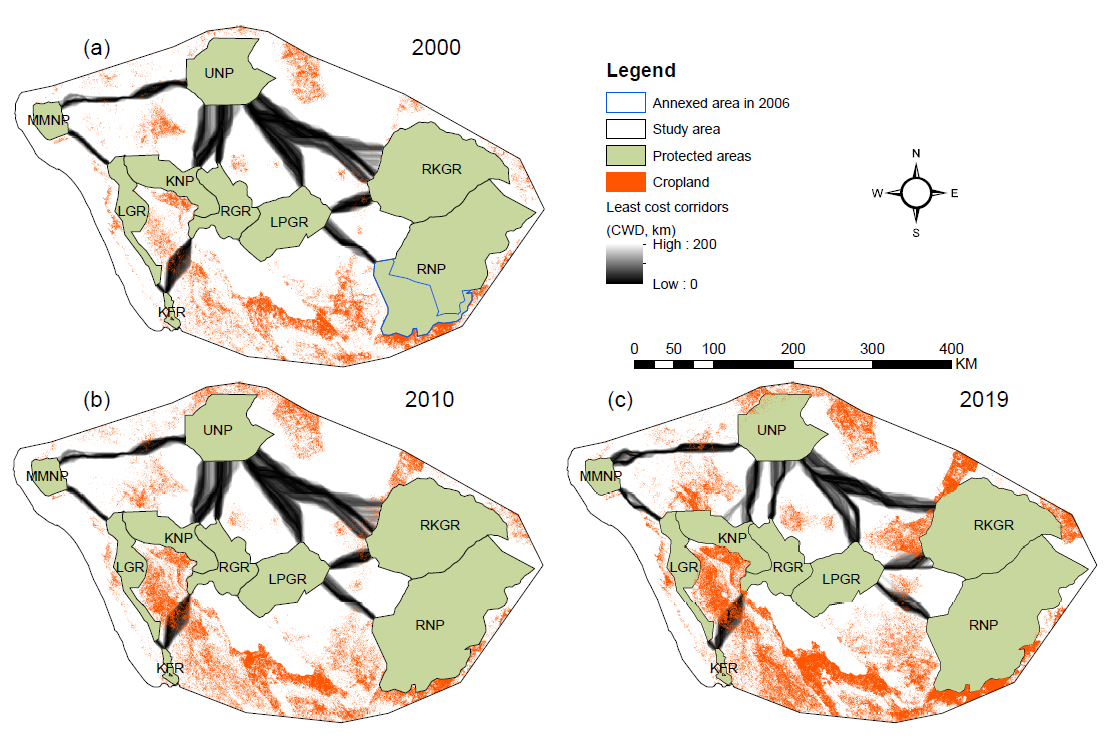

Supplement: S2 Fig — The polygon with the blue colour in (a) 2000 indicates area annexed by the RNP in 2006. (DOCX) [file pone.0292918.s002.docx]
