# Supplementary material for: Modelling elephant corridors over two decades reveals opportunities for conserving connectivity across a large protected area network
Source: PLoS One. 2023 Oct 13;18(10):e0292918. doi: 10.1371/journal.pone.0292918 (PMC10575508; doi:10.1371/journal.pone.0292918)
Supplement: S1 Table — (DOCX) [file pone.0292918.s003.docx]

S1 Table. Cross-tabulation error matrix for 2000, 2010 and 2019 land cover classification in south-western Tanzania.

| Land cover 2000 | Dense woodland | Open woodland | Burned area | Cropland | Water bodies | Total | User accuracy |
| --- | --- | --- | --- | --- | --- | --- | --- |
| Dense woodland | 118 | 2 | 0 | 0 | 0 | 120 | 0.983 |
| Open woodland | 1 | 117 | 2 | 0 | 0 | 120 | 0.975 |
| Burned area | 0 | 2 | 117 | 1 | 0 | 120 | 0.975 |
| Cropland | 0 | 0 | 2 | 118 | 0 | 120 | 0.983 |
| Water bodies | 0 | 0 | 0 | 0 | 120 | 120 | 1.000 |
| Total | 119 | 121 | 121 | 119 | 120 | 600 |  |
| Producer accuracy | 0.992 | 0.967 | 0.967 | 0.992 | 1.000 |  |  |
| Overall accuracy |  |  |  |  |  |  | 0.983 |
| Kappa |  |  |  |  |  |  | 0.979 |
|  |  |  |  |  |  |  |  |
| Land cover 2010 | Dense woodland | Open woodland | Burned area | Cropland | Water bodies | Total | User accuracy |
| Dense woodland | 117 | 3 | 0 | 0 | 0 | 120 | 0.975 |
| Open woodland | 2 | 114 | 3 | 1 | 0 | 120 | 0.950 |
| Burned area | 0 | 2 | 116 | 2 | 0 | 120 | 0.967 |
| Cropland | 0 | 2 | 2 | 116 | 0 | 120 | 0.967 |
| Water bodies | 0 | 0 | 0 | 0 | 120 | 120 | 1.000 |
| Total | 119 | 121 | 121 | 119 | 120 | 600 |  |
| Producer accuracy | 0.983 | 0.942 | 0.959 | 0.975 | 1.000 |  |  |
| Overall accuracy |  |  |  |  |  |  | 0.972 |
| Kappa |  |  |  |  |  |  | 0.965 |
|  |  |  |  |  |  |  |  |
| Land cover 2019 | Dense woodland | Open woodland | Burned area | Cropland | Water bodies | Total | User accuracy |
| Dense woodland | 117 | 3 | 0 | 0 | 0 | 120 | 0.975 |
| Open woodland | 2 | 114 | 3 | 1 | 0 | 120 | 0.950 |
| Burned area | 0 | 4 | 114 | 2 | 0 | 120 | 0.950 |
| Cropland | 0 | 1 | 3 | 116 | 0 | 120 | 0.967 |
| Water bodies | 0 | 0 | 0 | 1 | 119 | 120 | 0.992 |
| Total | 119 | 122 | 120 | 120 | 119 | 600 |  |
| Producer accuracy | 0.983 | 0.934 | 0.950 | 0.967 | 1.000 | 0.000 |  |
| Overall accuracy |  |  |  |  |  |  | 0.967 |
| Kappa |  |  |  |  |  |  | 0.958 |
