# Supplementary material for: Modelling elephant corridors over two decades reveals opportunities for conserving connectivity across a large protected area network
Source: PLoS One. 2023 Oct 13;18(10):e0292918. doi: 10.1371/journal.pone.0292918 (PMC10575508; doi:10.1371/journal.pone.0292918)
Supplement: S2 Table — (DOCX) [file pone.0292918.s004.docx]

S2 Table. Distribution of current flow density (Amps/cell) within the modelled corridors between PAs across three time steps (2000, 2010, and 2019) in south-western Tanzania.

| **PA** | | **Current flow density (Amps/cell)** | | | | | |
| --- | --- | --- | --- | --- | --- | --- | --- |
| **From** | **To** | **2000** | | **2010** | | **2019** | |
|  |  | **Min** | **Max** | **Min** | **Max** | **Min** | **Max** |
| MMNP | UNP | 0.04 | 0.13 | 0.04 | 0.15 | 0.03 | 0.20 |
| RGR | KFR | 0.03 | 0.14 | 0.33 | 0.15 | 0.03 | 0.16 |
| LGR | MMNP | 0.09 | 0.12 | 0.09 | 0.13 | 0.06 | 0.15 |
| LGR | KFR | 0.08 | 0.11 | 0.09 | 0.12 | 0.09 | 0.14 |
| LPGR | RNP | 0.03 | 0.11 | 0.04 | 0.12 | 0.04 | 0.13 |
| LPGR | RKGR | 0.04 | 0.10 | 0.05 | 0.11 | 0.02 | 0.12 |
| RGR | UNP | 0.02 | 0.09 | 0.02 | 0.10 | 0.02 | 0.12 |
| LPGR | UNP | 0.03 | 0.08 | 0.03 | 0.09 | 0.03 | 0.07 |
| KNP | UNP | 0.02 | 0.04 | 0.02 | 0.50 | 0.23 | 0.06 |
| UNP | RKGR | 0.01 | 0.03 | 0.01 | 0.04 | 0.03 | 0.05 |
